# Predicting dementia risk in primary care: development and validation of the Dementia Risk Score using routinely collected data

**DOI:** 10.1186/s12916-016-0549-y

**Published:** 2016-01-21

**Authors:** K. Walters, S. Hardoon, I. Petersen, S. Iliffe, R. Z. Omar, I. Nazareth, G. Rait

**Affiliations:** Research Department of Primary Care & Population Health, University College London, Rowland Hill St, London, NW3 2PF UK; Department of Statistical Science, University College London, Gower Street, London, WC1E 6BT UK

**Keywords:** Dementia, Primary care, Risk assessment, Routinely collected data

## Abstract

**Background:**

Existing dementia risk scores require collection of additional data from patients, limiting their use in practice. Routinely collected healthcare data have the potential to assess dementia risk without the need to collect further information. Our objective was to develop and validate a 5-year dementia risk score derived from primary healthcare data.

**Methods:**

We used data from general practices in The Health Improvement Network (THIN) database from across the UK, randomly selecting 377 practices for a development cohort and identifying 930,395 patients aged 60–95 years without a recording of dementia, cognitive impairment or memory symptoms at baseline. We developed risk algorithm models for two age groups (60–79 and 80–95 years). An external validation was conducted by validating the model on a separate cohort of 264,224 patients from 95 randomly chosen THIN practices that did not contribute to the development cohort. Our main outcome was 5-year risk of first recorded dementia diagnosis. Potential predictors included sociodemographic, cardiovascular, lifestyle and mental health variables.

**Results:**

Dementia incidence was 1.88 (95 % CI, 1.83–1.93) and 16.53 (95 % CI, 16.15–16.92) per 1000 PYAR for those aged 60–79 (n = 6017) and 80–95 years (n = 7104), respectively. Predictors for those aged 60–79 included age, sex, social deprivation, smoking, BMI, heavy alcohol use, anti-hypertensive drugs, diabetes, stroke/TIA, atrial fibrillation, aspirin, depression. The discrimination and calibration of the risk algorithm were good for the 60–79 years model; D statistic 2.03 (95 % CI, 1.95–2.11), C index 0.84 (95 % CI, 0.81–0.87), and calibration slope 0.98 (95 % CI, 0.93–1.02). The algorithm had a high negative predictive value, but lower positive predictive value at most risk thresholds. Discrimination and calibration were poor for the 80–95 years model.

**Conclusions:**

Routinely collected data predicts 5-year risk of recorded diagnosis of dementia for those aged 60–79, but not those aged 80+. This algorithm can identify higher risk populations for dementia in primary care. The risk score has a high negative predictive value and may be most helpful in ‘ruling out’ those at very low risk from further testing or intensive preventative activities.

**Electronic supplementary material:**

The online version of this article (doi:10.1186/s12916-016-0549-y) contains supplementary material, which is available to authorized users.

## Background

More than 115 million people are predicted to have dementia by 2050 [1], with huge associated health and social care costs [2]. There is both epidemiological [3, 4] and policy [5] support for the identification and management of modifiable risk factors for dementia to delay dementia onset. Around a third of Alzheimer’s disease cases might be attributable to potentially modifiable risk factors (diabetes, mid-life hypertension, mid-life obesity, depression, physical inactivity, smoking, low education) [3]. It has been estimated that a reduction in the seven main modifiable risk factors by 10–25 % would prevent an estimated 1–3 million dementia cases worldwide [4]. There is a strong drive internationally for clinicians to be more pro-active in dementia diagnosis [6, 7]. There is, however, a limited evidence base for current approaches to dementia screening and case-finding [8, 9] and further work needs to be completed to validate new methods across different settings, including primary care [9].

Many multi-factorial prognostic dementia risk models have been developed based on neuropsychological testing and sociodemographic, health, lifestyle, and environmental variables from a range of cohort studies, e.g. [10–20]. These have had variable discriminating power [10, 11], there is no one model that is recommended for population based settings [11], and none are widely used in practice. These risk scores entail collecting extra information from patients that would not form part of routine clinical care for the general population, for example, on fish oil intake [20], pesticide exposure [20], needing assistance with money or medication [19], years of education [12, 19, 20], depression symptom score [19, 20], genotype [12–14], or neuropsychological testing [13, 15, 17, 18], making these scores potentially more difficult and costly to implement to large populations in non-specialized clinical settings. One tool has recently been developed as a brief screening indicator to identify a high risk population for cognitive screening in primary care, using data from four cohort studies [19]. However, three of the seven factors in this tool are not routinely recorded in General Practitioner (GP) records in the United Kingdom (UK), and would have to be collected from patients individually. Validated risk scores developed using routinely collected primary care data have been used in practice for other disease areas, such as cardiovascular disease prediction, where they performed better than standard algorithms (e.g. Framingham) originally derived from cohort studies [21]. These scores can be easy to implement and calculated without collecting extra new information from the patient. They can be used to risk stratify an eligible practice population, as the process is automated and uses data already in medical records. No dementia risk model has yet been developed and validated using routinely collected primary care data in the general population. Our study objectives were to develop and validate a 5-year dementia risk score utilizing routinely collected data from a large nationally representative primary care database in the UK.

## Methods

### Study design

Cohort studies using routinely collected data; development and validation of a 5-year risk score for predicting newly recorded dementia diagnoses.

### Setting and data source

We used The Health Improvement Network (THIN) primary care database, which derives data from routine clinical practice in the UK [22]. Around 6 % of General Practices in the UK contribute data to the THIN database, which contains nearly 12 million patients and is broadly representative of the UK population [22, 23]. Data is collected longitudinally during routine care and includes consultations, symptoms, diagnoses, investigations, health measurements, prescriptions, surgical procedures, and referrals. Diagnoses from secondary care and other health information received by the practice are coded and entered using Read codes, a hierarchical coding system which maps onto ICD-10 codes, but which also includes symptom descriptions. THIN data is collected and anonymized centrally and linked by postal (zip) code for 150 households to population census data, including neighbourhood deprivation (quintiles of Townsend deprivation index) [24]. Diagnostic and prescribing information are generally well recorded and accurate [25, 26] and have been successfully used in numerous studies [22], including dementia [27–29]. Further, THIN data are subject to a range of quality assurance procedures [30, 31]. A validation study of dementia recording suggested a specificity of a GP recorded dementia diagnosis of 83 % and no false negatives in a small sample without recorded dementia [27].

We randomly selected 377 practices from 472 eligible practices providing acceptable quality data to THIN during our study period for a development cohort. The remaining 95 randomly selected eligible practices formed a completely separate validation cohort.

### Participants

**Fig. 1:**
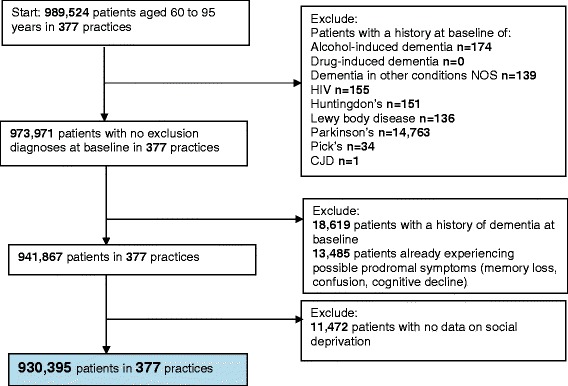
Flowchart of derivation of the development cohort

In both development and validation cohort studies we included individuals aged between 60 and 95 years contributing to the THIN database between January 1, 2000, and December 31, 2011. We excluded individuals with recorded dementia, cognitive impairment, memory symptoms and confusion prior to study entry, those with an exclusion diagnosis indicating specific sub-types of dementia syndrome (Parkinson’s disease, Huntingdon’s disease, Pick’s disease, alcohol-induced dementia, dementia in other conditions, Human Immunodeficiency Virus (HIV), Lewy body disease, Cruetzfeldt-Jacob Disease), and those with less than a year’s follow-up data, to allow time for patient history and risk factor information to be recorded (Fig. 1 and Additional file 1: Figure A1).

### Follow-up period

Follow-up time was restricted to a maximum of 5 years in both cohort studies. The start of follow-up was the latest of: 1) January 1, 2000; 2) when the individual turned 60 years; 3) one year following new registration with a THIN practice; 4) one year after the practice met standard criteria for accurate recording of deaths, consultation, health measurements, and prescribing [30, 31]. The end date was the earliest of dementia incident date, 5 years follow-up, patient died, patient developed an exclusion diagnosis (as listed above), patient left practice, practice left THIN database, or December 31, 2011.

### Main outcome

Newly recorded dementia diagnoses, including Alzheimer’s disease, vascular dementia, and unspecified or mixed dementia, but excluding dementia diagnoses associated with Parkinson’s disease, Lewy body dementia, Huntingdon, Picks, HIV, and drug-induced and alcohol-related dementia (Read code lists available from the authors) were the primary outcome.

### Risk factor measurements

Based on potential risk factors for dementia [3, 4, 32] available in THIN, we examined the following as predictor variables in the risk model:

1.  Sociodemographic measures: age (years), sex, social deprivation (quintiles of Townsend Index), calendar year at baseline (to account for temporal trends).
2.  Health status/lifestyle measurements: smoking status up to 5 years prior to baseline (current, non-smoker or ex-smoker), body mass index (BMI), lipids (total cholesterol/ high density lipoprotein (HDL) cholesterol ratio), systolic blood pressure (SBP), history of heavy alcohol use (more than 56 units per week for men/49 units per week for women), or a Read-code entry in their medical records indicating an alcohol problem.
3.  Medical diagnoses: diabetes, coronary heart disease (CHD), stroke/transient ischemic attack (TIA), or atrial fibrillation at any time prior to baseline. Current (in 12 months prior to baseline) depression diagnosis/treatment with antidepressant medication, anxiety diagnosis/treatment with anxiolytic medication.
4.  Prescription medication: As listed in (3) and anti-hypertensive drugs, hypnotic medication, statins, aspirin and other non-steroidal anti-inflammatory drugs (NSAIDs). Patients were identified as exposed to medications if they had received at least two consecutive prescriptions in the 12 months before baseline.

### Analysis

For both the development and validation cohort studies the study population was divided into two groups: those aged 60–79 years and aged 80–95 at baseline. At age 80 years, a sharp increased risk of dementia has previously been found [19], and in our population there were differences in the distribution of risk factors and their associations with dementia in those aged 60–79 years and older individuals. We considered additional stratification by sex but age-adjusted risk factor associations with dementia in men and women were similar, justifying combining both sexes in a single model. Separate model development was carried out for the two age groups in the development cohort and separate validation and calibration was performed for each age group in the validation cohort. Analyses were performed using Stata version 12.1.

### Sample size calculation

We conservatively estimated that 20 events were required per coefficient to fit a risk model based on studies evaluating the relationship between the number of events and the performance of a risk prediction model, which have shown that 15 events at least may be required to achieve a satisfactory level of model calibration [33]. There were a total of 25 coefficients for all the predictors initially considered, corresponding to 500 dementia events needed. Applying an inflation factor to adjust for clustering within practices of 10.741 for the 60–79 years age model (based on intra-class correlation coefficient of 0.00117, estimated from the data, and a mean cluster size of 2,122 people aged 60–79 years per practice), corresponded to a total of 500 × 10.741 = 5,371 dementia events. For the 80–95 years model, the inflation factor was 10.915 (based on intra-class correlation coefficient of 0.00863 and a mean cluster size of 346 people aged 80–95 years per practice), which corresponded to a total of 500 × 10.915 = 5,458 dementia events.

### Missing data imputation

We used the two-fold Fully Conditional Specification algorithm method for multiple imputation of longitudinal clinical datasets to impute missing data for both fixed (smoking and height) and time-varying variables (total cholesterol and HDL cholesterol, SBP and weight) in both the development and validation cohorts [34]. This algorithm is an efficient way to use the full longitudinal patient record rather than just the baseline measurements to inform the imputation. Missing data in the validation cohort was imputed separately from that in the development cohort. The remaining variables were complete. The imputation model included all variables in the analysis model, plus the outcome and cumulative hazard function. In the backwards elimination process, the variables were included in the final model if retained in 7 out of 10 imputed datasets to avoid over-selection of the variables [35].

### Development cohort: model development

For each age group (60–79 years and 80–95 years), we derived the dementia risk score using a Cox proportional hazards regression model, with robust standard errors to account for clustering of individuals within general practices. The assumption of proportional hazards was checked using plots of the log cumulative hazard function and Schoenfeld residuals. Continuous variables were centred and the assumption of a linear relationship was assessed using fractional polynomials, visual checks by plotting graphs of the log hazard ratio by increasing category of the continuous variable, and by inclusion of squared and cubic terms in the Cox models; transformations were made when linear relationships were not confirmed.

All variables were included in the full model prior to backwards elimination. We used backwards elimination to determine which variables should be retained, using the Akaike Information Criteria. After the elimination process we considered the interaction terms systolic blood pressure*anti-hypertensive medication and lipid ratio*statin prescriptions. Interactions were retained if significant and clinically meaningful.

### Validation cohort: validation and calibration

For each age group, the model developed using the development cohort was applied to the validation cohort, to assess performance. We assessed the discriminative performance of the dementia risk models by computing the Uno’s C [36] and Royston’s D [37] statistics for the validation cohort. Uno’s C and Royston’s D statistics were chosen as they have been shown to be less biased in the presence of censored data than other discriminative statistics [36, 37]. Each validation statistic was estimated separately for each imputed validation dataset, and then combined using Rubin’s rules to obtain an overall validation statistic. For Uno’s C statistic we calculated confidence intervals from bootstrapping. A random sub-sample of 15 % of the validation cohort was used as the vast size of the dataset made computation of bootstrap confidence intervals for the full sample unfeasible. We assessed calibration by comparing the observed and predicted dementia risk in the validation cohort per decile of predicted risk, and computing the calibration slope. We calculated the sensitivity, specificity, positive predictive value (PPV), and negative predictive value (NPV) using a range of potential risk thresholds, to explore the clinical utility of the risk algorithms.

## Results

### Development cohort study

We identified 930,395 eligible patients aged 60–95 years in 377 practices in the development cohort study, of which 800,013 were aged 60–79 years and 130,382 aged 80–95 years at baseline (Fig. 1).

#### Development cohort aged 60–79 years

##### Baseline characteristics

There were 413,974 (52 %) women in the 60–79 years development cohort, the mean age at baseline was 65.6 years (SD 6.1 years; Table 1). Missing data on health measurements are detailed in Additional file 1: Table A.1, with characteristics after multiple imputation in Table A.2.

**Table 1:** Characteristics of development and validation cohorts for those aged 60–79 years (before multiple imputation)

	Development cohort n = 800,013 Median follow-up 5 years (IQR, 3.15–5 years) Dementia events n = 6,017			Validation cohort n = 226,140 Median follow-up 5 years (IQR, 3.27–5 years) Dementia events n = 1,699		
	Obs	Mean	SD	Obs	Mean	SD
Baseline age, years	800,013	65.6	6.08	226,140	65.6	6.11
Baseline total cholesterol, mmol/L	242,045	5.21	1.13	64,832	5.25	1.13
Baseline HDL cholesterol, mmol/L	167,937	1.42	0.41	45,682	1.42	0.42
Baseline weight, kg	226,671	79.2	16.9	59,594	79.1	16.8
Baseline systolic blood pressure, mmHg	452,306	142.4	17.0	125,744	142.7	17.1
Height, m	553,195	1.67	0.10	159,136	1.67	0.10
Baseline BMI, kg/m^2^	193,524	28.5	5.44	51,590	28.4	5.36
Baseline lipid ratio	166,420	3.88	1.17	45,352	3.91	1.17
	Obs	Freq	%	Obs	Freq	%
Sex	800,013			226,140		
Men		386,039	48.3		109,108	48.3
Women		413,974	51.8		117,032	51.8
Local area deprivation score (quintiles)	800,013			226,140		
1 (=least deprived)		218,198	27.3		71,040	31.4
2		194,637	24.3		57,763	25.5
3		166,956	20.9		42,278	18.7
4		134,103	16.8		33,945	15.0
5 (=most deprived)		86,119	10.8		21,114	9.3
Baseline smoking status	756,115			213,419		
Never		323,345	42.8		96,256	45.1
Past		286,763	37.9		78,608	36.8
Current		146,007	19.3		38,555	18.1
History of very heavy drinking/alcohol problem	800,013	22,308	2.8	226,140	6,011	2.7
History of diabetes	800,013	70,377	8.8	226,140	18,662	8.3
History of coronary heart disease	800,013	93,408	11.7	226,140	26,016	11.5
History of stroke or transient ischemic attack	800,013	38,976	4.9	226,140	10,930	4.8
History of atrial fibrillation	800,013	24,763	3.1	226,140	7,085	3.1
Depression or use of anti-depressants at baseline	800,013	83,464	10.4	226,140	23,583	10.4
Anxiety or use of anxiolytics at baseline	800,013	29,690	3.7	226,140	8,549	3.8
Use of anti-hypertensive drugs at baseline	800,013	274,657	34.3	226,140	75,359	33.3
Use of statins at baseline	800,013	151,275	18.9	226,140	39,738	17.6
Use of hypnotics at baseline	800,013	30,787	3.9	226,140	8,736	3.9
Use of NSAIDs (other than aspirin) at baseline	800,013	98,397	12.3	226,140	27,546	12.2
Use of aspirin at baseline	800,013	127,550	15.9	226,140	34,756	15.4

##### Incidence of dementia

In the development cohort there were 6,017 new diagnoses in 800,013 individuals with 3,205,190 Person Years at Risk (PYAR), corresponding to a crude overall incidence of dementia of 1.88/1000 PYAR (95 % CI, 1.83–1.93) for 60–79 year olds. This included 1,831 newly recorded diagnoses of Alzheimer’s dementia, 1,308 of vascular dementia and 2,878 of unspecified or mixed dementia during follow-up.

##### Associations of risk factors with new GP recorded dementia diagnoses within 5 years

Newly recorded dementia diagnoses were associated with increasing age, female sex, calendar year, and living in a deprived area (Additional file 1: Table A.3). There were positive associations with current smoking, hazardous/harmful alcohol drinking, and history of stroke/TIA, diabetes, CHD, atrial fibrillation, or current depression/anti-depressant drug, anxiety/anxiolytic drug, hypnotic drug, and aspirin use. There were no significant associations with NSAIDs (excluding aspirin) and anti-hypertensive drugs. There was a small negative association with both BMI and systolic blood pressure.

##### Selection of variables for risk model

Following backwards elimination, age, sex, deprivation, calendar year, BMI, current anti-hypertensive use, smoking status, hazardous/harmful alcohol drinking, current depression, current aspirin use, and history of diabetes, stroke, TIA and atrial fibrillation were all retained in the model (Table 2). Because statin use, lipid ratio, and SBP were all eliminated in the backwards elimination, interaction terms for statin use*lipid ratio and anti-hypertensive use*SBP were not considered.

**Table 2:** Final dementia risk model for cohort aged 60–79 years after backwards elimination (from development cohort)

	Coefficient^a^	95 % CI	HR^a^	95 % CI
Age, per year increase	0.209	0.200 to 0.219	1.23	1.22 to 1.25
Age^2^, per unit increase	−0.003	−0.004 to −0.003	0.997	0.996 to 0.997
Gender (female vs. male)	0.129	0.074 to 0.183	1.14	1.08 to 1.20
Calendar year, per year increase	0.045	0.035 to 0.054	1.05	1.04 to 1.06
Local area deprivation score (quintile)				
1 (=least deprived)	0		1	
2	0.013	−0.063 to 0.090	1.01	0.94 to 1.09
3	0.118	0.041 to 0.194	1.13	1.04 to 1.22
4	0.202	0.123 to 0.280	1.22	1.13 to 1.32
5 (=most deprived)	0.226	0.138 to 0.314	1.25	1.15 to 1.37
BMI (kg/m^2^), per unit increase	−0.062	−0.069 to −0.054	0.94	0.93 to 0.95
BMI^2^, per unit increase	0.003	0.002 to 0.003	1.003	1.002 to 1.003
Current anti-hypertensive use (yes vs. no)	−0.132	−0.190 to −0.074	0.88	0.83 to 0.93
Smoking status				
Never	0		1	
Past	−0.068	−0.127 to −0.009	0.93	0.88 to 0.99
Current	−0.087	−0.168 to −0.005	0.92	0.85 to 1.00
History of alcohol problem (yes vs. no)	0.444	0.287 to 0.600	1.56	1.33 to 1.82
History of diabetes (yes vs. no)	0.287	0.205 to 0.368	1.33	1.23 to 1.45
Current depression/use of anti-depressants (yes vs. no)	0.834	0.770 to 0.897	2.30	2.16 to 2.45
History of stroke or transient ischemic attack (yes vs. no)	0.577	0.500 to 0.654	1.78	1.65 to 1.92
History of atrial fibrillation (yes vs. no)	0.221	0.120 to 0.322	1.25	1.13 to 1.38
Current aspirin use (yes vs. no)	0.253	0.189 to 0.316	1.29	1.21 to 1.37

^a^Coefficients and hazard ratios (HRs) are obtained by building Cox models separately within each of the 10 imputation datasets and then combining the results using Rubin’s rules. Baseline 5-year survival function, So(5) = 0.9969

Age^2^ = age-squared i.e. the hazard ratio corresponds to the relative increase in hazard per unit increase in the quadratic function of age

#### Development cohort aged 80–95 years

##### Baseline characteristics

There were 86,096 (66 %) women in the 80–95 years development cohort, with a mean age at baseline of 85 years (SD 3.9 years; Table 3). Missing data on health measurements are reported in Additional file 1: Table A.1. Characteristics after multiple imputation are reported in Table A.4.

**Table 3:** Characteristics of development and validation cohorts for those aged 80–95 years (before imputation)

	Development cohort n = 130,382 Median follow-up 3.76 years (IQR, 1.71–5 years) Dementia events n = 7,104			Validation cohort n = 38,084 Median follow-up 3.92 years (IQR, 1.75–5 years) Dementia events n = 1,923		
	Obs	Mean	SD	Obs	Mean	SD
Baseline age, years	130,382	84.8	3.93	38,084	84.9	3.97
Baseline total cholesterol, mmol/L	26,841	4.99	1.19	6,785	5.08	1.21
Baseline HDL cholesterol, mmol/L	16,630	1.50	0.44	4,066	1.49	0.44
Baseline weight, kg	31,272	67.1	14.0	8,038	67.2	13.8
Baseline systolic blood pressure, mmHg	78,979	146.9	19.8	22,347	147.5	19.8
Height, m	62,622	1.62	0.10	17,616	1.62	0.10
Baseline BMI, kg/m^2^	24,091	25.7	4.65	6,213	25.7	4.53
Baseline lipid ratio	16,566	3.49	1.10	4,054	3.57	1.13
	Obs	Freq	%	Obs	Freq	%
Sex	130,382			38,084		
Men		44,286	34.0		13,017	34.2
Women		86,096	66.0		25,067	65.8
Local area deprivation score (quintiles)	130,382			38,084		
1 (=least deprived)		26,643	20.4		10,048	26.4
2		30,143	23.1		9,307	24.4
3		28,970	22.2		7,830	20.6
4		26,758	20.5		6,644	17.5
5 (=most deprived)		17,868	13.7		4,255	11.2
Baseline smoking status	113,391			32,702		
Never		63,684	56.2		19,389	59.3
Past		39,778	35.1		10,697	32.7
Current		9,929	8.8		2,616	8.0
History of very heavy drinking/alcohol problem	130,382	921	0.7	38,084	250	0.7
History of diabetes	130,382	12,762	9.8	38,084	3,331	8.8
History of coronary heart disease	130,382	28,190	21.6	38,084	8,281	21.7
History of stroke or transient ischemic attack	130,382	20,221	15.5	38,084	5,824	15.3
History of atrial fibrillation	130,382	14,518	11.1	38,084	4,293	11.3
Depression or use of anti-depressants at baseline	130,382	17,201	13.2	38,084	4,886	12.8
Anxiety or use of anxiolytics at baseline	130,382	5,953	4.6	38,084	1,816	4.8
Use of anti-hypertensive drugs at baseline	130,382	58,323	44.7	38,084	16,396	43.1
Use of statins at baseline	130,382	16,546	12.7	38,084	4,111	10.8
Use of hypnotics at baseline	130,382	14,121	10.8	38,084	4,137	10.9
Use of NSAIDs (other than aspirin) at baseline	130,382	15,056	11.6	38,084	4,430	11.6
Use of aspirin at baseline	130,382	41,448	31.8	38,084	11,830	31.1

##### Incidence of dementia

In the 80–95 years development cohort there were 1,483 newly recorded diagnoses of Alzheimer’s dementia, 1,331 of vascular dementia, and 4,290 of unspecified or mixed dementia during follow-up. In total, there were 7,104 new diagnoses in 429, 670 PYAR, corresponding to a crude incidence of dementia of 16.53/1000 PYAR (95 % CI, 16.15–16.92) for those aged 80–95 years at baseline.

##### Associations of risk factors with new GP recorded dementia diagnosis within 5 years

New dementia diagnoses were associated with increasing age and female sex (Additional file 1: Table A.3). There were positive associations with history of stroke/TIA, diabetes, atrial fibrillation, statin prescriptions, hazardous/harmful alcohol drinking, current depression/anti-depressant drugs, anxiety/anxiolytic drugs, hypnotic drugs and aspirin use. There were no significant associations with living in a deprived area, CHD, and total cholesterol/HDL ratio. There was a small negative association with current smoking, BMI, systolic blood pressure, anti-hypertensive drugs, and NSAIDs (excluding aspirin).

##### Selection of variables for risk model

Following backwards elimination, age, sex, calendar year, BMI, current anti-hypertensive use, SBP, lipid ratio, smoking status, hazardous/harmful alcohol drinking, current depression/anti-depressants, current anxiety/anxiolytics, current aspirin use, current other NSAID use, and history of diabetes, stroke, or TIA and atrial fibrillation were all retained in the model (Table 4). As statin use was excluded, the interaction term statin use*lipid ratio was not considered. An interaction term for SBP*anti-hypertensive use was considered, but was not statistically significant (*P* = 0.6) and therefore was not included.

**Table 4:** Final dementia risk model for cohort aged 80–95 years after backwards elimination (development cohort)

	Coefficient^a^	95 % CI	HR^a^	95 % CI
Age, per year increase	0.055	0.047 to 0.062	1.06	1.05 to 1.06
Age^2^, per unit increase	−0.005	−0.007 to −0.004	0.995	0.993 to 0.996
Gender (female v male)	0.160	0.104 to 0.216	1.17	1.11 to 1.24
Calendar year, per year increase	0.074	0.063 to 0.084	1.08	1.07 to 1.09
BMI (kg/m^2^), per unit increase	−0.050	−0.063 to-0.036	0.95	0.94 to 0.96
Current anti-hypertensive use (yes vs. no)	−0.249	−0.301 to −0.197	0.78	0.74 to 0.82
Systolic blood pressure (mmHg), per unit increase	−0.006	−0.008 to −0.005	0.994	0.992 to 0.995
Lipid ratio (per unit increase)	0.042	−0.055 to 0.138	1.04	0.95 to 1.15
Smoking status				
Never	0		1	
Past	−0.178	−0.233 to −0.122	0.84	0.79 to 0.89
Current	−0.134	−0.229 to −0.039	0.88	0.80 to 0.96
History of alcohol problem (yes vs. no)	0.256	−0.009 to 0.521	1.29	0.99 to 1.68
History of diabetes (yes vs. no)	0.183	0.102 to 0.264	1.20	1.11 to 1.30
History of stroke or transient ischemic attack (yes vs. no)	0.242	0.177 to 0.306	1.27	1.19 to 1.36
History of atrial fibrillation (yes vs. no)	0.057	−0.018 to 0.132	1.06	0.98 to 1.14
Current depression/use of anti-depressants (yes vs. no)	0.400	0.335 to 0.465	1.49	1.40 to 1.59
Current anxiety/use of anxiolytics (yes vs. no)	0.136	0.034 to 0.237	1.15	1.04 to 1.27
Current NSAID use, excluding aspirin (yes vs. no)	−0.157	−0.237 to −0.078	0.86	0.79 to 0.93
Current aspirin use (yes vs. no)	0.092	0.037 to 0.147	1.10	1.04 to 1.16

^a^Coefficients and hazard ratios (HRs) are obtained by building Cox models separately within each of the 10 imputation datasets and then combining the results using Rubin’s rules. Baseline 5 year survival function, So(5) = −0.9277

Age^2^ = age-squared i.e. the hazard ratio corresponds to the relative increase in hazard per unit increase in the quadratic function of age

### Validation cohort study

We identified 264,224 eligible patients aged 60–95 years in 95 practices for the validation cohort, of which 226,140 were aged 60–79 years and 38,084 were aged 80–95 years at baseline (Additional file 1: Figure A.1).

#### Validation cohort aged 60–79 years

##### Baseline characteristics/incidence of dementia

The characteristics of the validation cohort were similar to the development cohort (Table 1). Missing data on health measurements are reported in Additional file 1: Table A.1, with characteristics after multiple imputation in Table A.2. Incidence rates for dementia were similar to those in the development cohort, with 1,699 new diagnoses in 226,140 individuals with 915,380 PYAR, corresponding to a crude overall incidence of dementia of 1.86/1000 PYAR (95 % CI, 1.77–1.95) for 60–79 year olds. This included 528 newly recorded diagnoses of Alzheimer’s dementia, 384 of vascular dementia, and 787 of unspecified or mixed dementia during follow-up.

##### Discrimination and calibration

The model performed well in terms of discrimination, with a Royston’s D statistic of 2.03 (95 % CI, 1.95–2.11) and Uno’s C index 0.84 (95 % CI, 0.81–0.87). The calibration slope suggested good calibration (0.98, 95 % CI, 0.93–1.02).

##### Risk classification

Utilizing a range of possible cut-offs to indicate ‘high risk’ for dementia, the specificity of the risk algorithm was high but with lower sensitivity, and there was a high NPV, but a low PPV (Table 5). For example, if we chose a threshold to define high risk of 2 %, the specificity would be 85.15 %, sensitivity 58.36 %, PPV 2.89, and NPV 99.63. We include details of how to calculate the risk of dementia for a new patient in Additional file 1.

**Table 5:** Risk classification using the 60–79 years dementia risk algorithm when applied to validation cohort

Cut off for high risk	Sensitivity	Specificity	PPV	NPV	Patients classified as high risk, n (%)	Patients classified as high risk who develop dementia, n (%)	Patients classified as low risk, n (%)	Patients classified as low risk who develop dementia, n (%)
1 %	77.70	73.05	2.14	99.77	61,803 (27.33)	1,320 (2.14)	164,337 (72.67)	379 (0.23)
2 %	58.36	85.15	2.89	99.63	34,323 (15.18)	992 (2.89)	191,817 (84.82)	707 (0.37)
5 %	19.39	97.03	4.71	99.38	6,989 (3.09)	329 (4.71)	219,151 (96.91)	1,370 (0.62)
10 %	5.62	99.52	8.20	99.29	1,164 (0.51)	95 (8.2)	224,976 (99.49)	1,604 (0.71)
20 %	0.72	99.96	10.87	99.25	113 (0.05)	12 (10.87)	226,027 (99.95)	1,687 (0.75)

PPV, Positive predictive value; NPV, Negative predictive value

#### Validation cohort aged 80–95 years

##### Baseline characteristics/incidence of dementia

The characteristics of those aged 80–95 years in the validation cohort were similar to the development cohort (Table 3). Missing data on health measurements are reported in Additional file 1: Table A.1, with characteristics after multiple imputation in Table A.3. Incidence rates for dementia were similar to those in the development cohort, with 1,923 new diagnoses in 38,084 individuals with 127,510 PYAR, corresponding to a crude overall incidence of dementia of 15.08/1000 PYAR (95 % CI, 14.42–15.77) for 80–95 year olds. This included 408 newly recorded diagnoses of Alzheimer’s dementia, 364 of vascular dementia, and 1,151 of unspecified or mixed dementia during follow-up.

##### Discrimination and calibration

The model from the development cohort performed poorly in terms of discrimination (Royston’s D statistic 0.86, 95 % CI, 0.76–0.95 and Uno’s C index 0.56, 95 % CI, 0.55–0.58) and calibration (calibration slope 1.04, 95 % CI, 0.89–1.18) when applied to the validation cohort. As this model performed poorly we have not reported on risk classification.

## Discussion

This study developed risk algorithms for predicting a new recorded dementia diagnosis in two age groups in primary care. In our validation study, the dementia risk algorithm developed for the 60–79 year old population performed well, but the algorithm for the older 80–95 years population did not. Our model is the first to be derived entirely from routinely collected health data, which can be calculated without collecting additional information from the patient. In people aged between 60–79 years, the dementia risk score included records of depression, stroke, high alcohol consumption, diabetes, atrial fibrillation, aspirin use, smoking, decreasing weight, and untreated blood pressure. Aspirin use may be a marker for underlying vascular risk. The directions of associations of some factors, such as weight and cholesterol, have been shown to change in later life with the onset of disability, frailty and cognitive decline and potential pre-clinical dementia [38, 39]. In our study, the ‘high risk’ population may include those with pre-clinical or undetected/recorded dementia, which may explain some of the associations observed with individual factors. Our algorithm uses routinely collected healthcare data to predict the risk of a GP recorded diagnosis within 5 years, and the profile of risk factors within the score is different to those aimed at identifying future risk, for example mid-life risk scores for dementia [40].

At a low threshold of 1 %, our risk algorithm had a sensitivity of 78 % and specificity of 73 %. With thresholds of 2 % or above, our risk algorithm had higher specificity (85 %) but a correspondingly lower sensitivity (58 %). In previous prediction models derived from cohort studies, models have generally had either high specificity with low sensitivity or vice versa [10, 11], and the choice of threshold will depend on the intended use.

### Strengths and limitations

Our development cohort study included more than 900,000 older people from across the UK registered with THIN General Practices, with more than 13,000 new dementia events recorded. The findings are likely to be generalizable to the UK population, but may not be generalizable to other different healthcare settings. The data source includes longitudinal data on a wide range of potential risk factors, including demographic factors, lifestyle, heath status measurements, medical history/diagnoses, and drugs. We had power to consider a wide range of potentially important risk factors, in comparison to cohort studies with smaller samples [10–20]. In those aged 60–79 years, we had good recording of data for most factors, and for missing data at baseline we used robust multiple imputation techniques utilizing the entire patient record, taking into account the longitudinal records rather than relying solely on baseline parameters.

Using routinely collected data to develop the risk algorithm has some inherent limitations. It may be less complete in terms of potential predictor variables than cohorts designed for research. The older cohort (80–95 years) had fewer routine measurements of health status such as BMI and lipid profile. The current validation applies to use of the risk score in the case where the GP has complete information on the factors in the model. There were low levels of missing data in some individuals on smoking status and BMI for those 60–79 years, which we imputed for our analysis. For all other factors in the final model, if missing, the factor was presumed to be absent.

Some potential risk factors, such as family history of dementia, physical activity or educational status, are poorly recorded in routine UK primary care and could not be included. Studies suggest that chronic and significant medical diagnoses entered in electronic records are likely to be accurate [25]. Other evidence suggests dementia is under-recorded in primary care [41]. Our incidence rates for dementia were lower than rates reported in studies using screening, particularly for those over 80 years [42]; however, there is some evidence that dementia prevalence is stabilizing more recently, despite population ageing [43], and our study is based on more contemporary data. This potential under-recording of dementia diagnoses in GP records may lead to an underestimation of the true predictive power of the risk score. In common with most risk models, we only accounted for baseline variables and for time-varying factors, exposure status may change during the follow-up period. Routinely collected data has the advantage of reflecting the data normally available to a clinician in practice.

### Implications

We used routinely collected primary care data to derive a relatively simple new risk algorithm, predicting a new GP recorded dementia diagnosis within 5 years, which worked well in those aged 60–79 years, but not in older age groups. This supports the previous suggestion that given the steep rise in risk of dementia at 80 years, it would be reasonable to test for dementia beyond this point on the basis of age alone [19]. It is likely that risk scores using traditional risk factors will not perform well in this population, and a different approach might be needed to identify a higher risk group aged 80 or above using, for example, measures of frailty.

Our new dementia risk algorithm for 60–79 year olds can be added to clinical software systems and a practice could, for example, run this risk model on all eligible people and offer those at risk more detailed testing or specific preventive management. Using a range of thresholds, there was good specificity but lower sensitivity, and a very high NPV but a low PPV. This risk algorithm may be most helpful to ‘rule out’ those at low risk from dementia case finding programs. This might avoid unnecessary investigations and anxiety for those at very low risk and make these programs more cost-effective. The risk algorithm may enable the identification of ‘at risk’ groups to approach for future research studies. We report a range of thresholds to allow clinicians or researchers to select the threshold that gives the optimum balance of sensitivity and specificity for dementia risk, depending on the intended use.

Further research should be undertaken to explore the performance of the Dementia Risk Score in different settings and populations, including variations in performance in areas where the prevalence, detection, and recording of dementia by GPs is very low or very high. We also need to further understand how the tool might be used in practice, the ethical implications, and what the impact of this might be for older people, clinicians, and the potential costs for health services.

## Conclusion

Routinely collected health data can predict five year risk of recorded diagnosis of dementia in primary care for individuals aged 60-79 years, but not for those aged 80 years or more. This risk score can be used to identify higher risk populations for dementia in primary care. The risk score has a high negative predictive value and may be most helpful in ‘ruling out’ those at very low risk from further testing.

### Availability of data and materials

Codelists, Do-files to construct the Dementia Risk Score, and data are available from the corresponding author, Dr. Kate Walters.

### Ethics

The NHS South-East Multi-centre Research Ethics Committee approved the scheme for THIN to provide anonymous patient data to researchers. Scientific approval for this study was obtained from THIN Scientific Review Committee in October 2012.

## Additional file

Additional file 1:
**Supplementary tables** (DOCX 46 kb)

## Abbreviations

BMI: Body mass index
CHD: Coronary Heart Disease
GP: General Practitioner
HDL: High Density Lipoprotein
NSAIDS: Non-steroidal anti-inflammatory drugs
NPV: Negative predictive value
PPV: Positive predictive value
PYAR: Person years at risk
THIN: The Health Improvement Network primary care database
SBP: Systolic blood pressure
TIA: Transient ischemic attack
