# Supplementary material for: Predicting dementia risk in primary care: development and validation of the Dementia Risk Score using routinely collected data
Source: BMC Med. 2016 Jan 21;14:6. doi: 10.1186/s12916-016-0549-y (PMC4722622; doi:10.1186/s12916-016-0549-y)
Supplement: Additional file 1: — Supplementary tables (DOCX 46 kb) [file 12916_2016_549_MOESM1_ESM.docx]

**“Predicting dementia risk in primary care: development and validation of the Dementia Risk Score (DRS) using routinely collected data.”**

**Appendix A: Supplementary tables**

**Table A.1:** Missing data in time-varying predictor variables for multiple imputation.

| **Aged 60-79 years** | **Development cohort** | |  |  | **Validation cohort** | |  |  |
| --- | --- | --- | --- | --- | --- | --- | --- | --- |
|  | **% of patients** | |  |  | **% of patients** | |  |  |
| **Number of imputation years with data** | **SBP** | **Total cholesterol** | **HDL cholesterol** | **Weight** | **SBP** | **Total cholesterol** | **HDL cholesterol** | **Weight** |
| 0 (= no data at any time) | 12.7 | 33.7 | 43.0 | 34.1 | 12.9 | 35.3 | 43.5 | 36.8 |
| 1 | 15.8 | 19.3 | 19.8 | 23.4 | 16.3 | 19.4 | 19.7 | 23.4 |
| 2 | 15.3 | 13.9 | 13.1 | 15.4 | 15.3 | 13.5 | 12.8 | 14.8 |
| 3 | 12.6 | 10.4 | 9.1 | 10.2 | 12.6 | 10.2 | 9.0 | 9.5 |
| 4 | 12.0 | 8.9 | 6.9 | 7.3 | 11.4 | 8.5 | 6.9 | 6.5 |
| 5 | 12.1 | 7.6 | 5.0 | 5.3 | 11.9 | 7.3 | 5.0 | 4.9 |
| 6 (= data in every imputation year) | 19.5 | 6.2 | 3.1 | 4.4 | 19.6 | 6.0 | 3.1 | 4.2 |
| **Data in at least one imputation year** | **87.3** | **66.3** | **57.0** | **65.9** | **87.1** | **64.7** | **56.5** | **63.2** |
| **Numbers of patients with data in at least one imputation year** | **698571** | **530249** | **456247** | **527289** | **197058** | **146403** | **127679** | **142875** |
| **Aged 80-95 years** | **Development cohort** | |  |  | **Validation cohort** | |  |  |
|  | **% of patients** | |  |  | **% of patients** | |  |  |
| **Number of imputation years with data** | **SBP** | **Total cholesterol** | **HDL cholesterol** | **Weight** | **SBP** | **Total cholesterol** | **HDL cholesterol** | **Weight** |
| 0 (= no data at any time) | 16.8 | 52.8 | 64.4 | 50.4 | 17.5 | 56.7 | 67.2 | 55.1 |
| 1 | 16.4 | 17.2 | 14.7 | 20.3 | 16.3 | 15.8 | 13.6 | 19.0 |
| 2 | 15.4 | 11.0 | 8.8 | 11.7 | 15.0 | 10.1 | 7.6 | 10.4 |
| 3 | 12.7 | 7.4 | 5.2 | 7.3 | 12.8 | 6.8 | 5.1 | 6.5 |
| 4 | 12.0 | 5.4 | 3.5 | 5.0 | 11.7 | 5.0 | 3.3 | 4.0 |
| 5 | 11.3 | 3.7 | 2.2 | 3.2 | 11.3 | 3.4 | 2.0 | 3.0 |
| 6 (= data in every imputation year) | 15.4 | 2.5 | 1.2 | 2.1 | 15.4 | 2.3 | 1.1 | 2.0 |
| **Data in at least one imputation year** | **83.2** | **47.3** | **35.6** | **49.6** | **82.5** | **43.4** | **32.8** | **44.9** |
| **Numbers of patients with data in at least one imputation year** | **108530** | **61605** | **46390** | **64643** | **31408** | **16509** | **12499** | **17092** |

Missing data were imputed using the two-fold fully Conditional Specification (FCS) algorithm, which incorporates data on time-varying predictors in years before and after the baseline (6 imputation years in total) to inform the baseline imputations. Height and smoking status also had missing data. These variables were assumed fixed for the purposes of imputation and data only at baseline on these variables was used in the imputations. The numbers (%) of patients with smoking status data at baseline were 756115 (94.5%) and 213419 (94.4%) in the 60-79 years development and validation cohorts respectively. The numbers (%) of patients with height data at baseline were 553195 (69.1%) and 159136 (70.4%) in the 60-79 years development and validation cohorts respectively. The numbers (%) of patients with smoking status data at baseline were 113391 (87.0%) and 32702 (85.9%) in the 80-95 years development and validation cohorts respectively. The numbers (%) of patients with height data at baseline were 62622 (48.0%) and 17616 (46.3%) in the 80-95 years development and validation cohorts respectively.

**Table A.2: Characteristics of sample aged 60-79 years after imputation for variables with missing data at baseline.**

|  | **Development cohort**  **(n = 800,013)** | | | **Validation cohort**  **(n = 226,140)** | | |
| --- | --- | --- | --- | --- | --- | --- |
|  | **Mean** | **sd** | **Mean** | | **sd** |  |
|  |  |  |  | |  |  |
| **Baseline Total cholesterol, mmol/L** | 5.58 | 1.10 | 5.61 | | 1.08 |  |
| **Baseline HDL cholesterol, mmol/L** | 1.48 | 0.42 | 1.48 | | 0.42 |  |
| **Baseline Weight, kg** | 76.7 | 15.9 | 76.5 | | 15.7 |  |
| **Baseline Systolic blood pressure, mmHg** | 141.4 | 17.1 | 141.6 | | 17.1 |  |
| **Height, m** | 1.67 | 0.10 | 1.67 | | 0.10 |  |
| **Baseline BMI, kg/m2** | 27.5 | 4.98 | 27.3 | | 4.88 |  |
| **Baseline lipid ratio** | 4.00 | 1.17 | 4.02 | | 1.17 |  |
|  |  |  |  | |  |  |
|  | **Frequency** | **%** | **Frequency** | | **%** |  |
|  |  |  |  | |  |  |
| **Baseline smoking status** |  |  |  | |  |  |
| Never | 342,934 | 42.9 | 102,213 | | 45.2 |  |
| Ex | 301,133 | 37.6 | 82,645 | | 36.5 |  |
| Current | 155,946 | 19.5 | 41,282 | | 18.3 |  |

**Table A.3: Age and gender adjusted associations of each predictor variable with dementia in development cohorts**

|  | **Cohort aged 60-79 years** |  | **Cohort aged 80+**  **years** |  |
| --- | --- | --- | --- | --- |
|  | **HR* (95%CI)** | **p-value** | **HR* (95% CI)** | **p-value** |
| **Age, per year increase** | 1.23 (1.22 to 1.24) | <0.001 | 1.08 (1.07 to 1.08) | <0.001 |
| **Age^2^, per unit increase** | 0.998 (0.997 to 0.998) | <0.001 | 0.995 (0.994 to 0.997) | <0.001 |
| **Gender (female v male)** | 1.19 (1.13 to 1.25) | <0.001 | 1.23 (1.16 to 1.29) | <0.001 |
| **Calendar year, per year increase** | 1.05 (1.04 to 1.06) | <0.001 | 1.08 (1.07 to 1.09) | <0.001 |
| **Townsend local area deprivation quintile** |  | <0.001 |  | 0.6 |
| **1 (=least deprived)** | 1 |  | 1 |  |
| **2** | 1.03 (0.95 to 1.11) |  | 1.00 (0.93 to 1.07) |  |
| **3** | 1.15 (1.06 to 1.24) |  | 0.97 (0.90 to 1.04) |  |
| **4** | 1.26 (1.17 to 1.36) |  | 1.02 (0.95 to 1.10) |  |
| **5 (=most deprived)** | 1.34 (1.23 to 1.46) |  | 0.97 (0.89 to 1.05) |  |
| **BMI (kg/m2), per unit increase** | 0.95 (0.94 to 0.95) | <0.001 | 0.95 (0.94 to 0.96) | <0.001 |
| **BMI^2^, per unit increase** | 1.003 (1.002 to 1.003) | <0.001 | - | - |
| **Current anti-hypertensive use (yes v no)** | 1.04 (0.99 to 1.10) | 0.1 | 0.85 (0.81 to 0.89) | <0.001 |
| **SBP (mmHg), per unit increase** | 0.994 (0.992 to 0.997) | <0.001 | 0.989 (0.987 to 0.992) | <0.001 |
| **Anti-hypertensive X SBP** | 0.997 (0.994 to 1.001) | 0.1 | 1.000 (0.997 to 1.003) | 0.9 |
| **Current statin use (yes v no)** | 1.26 (1.17 to 1.34) | <0.001 | 1.16 (1.06 to 1.26) | <0.001 |
| **Lipid ratio, per unit increase** | 0.93 (0.88 to 0.98) | 0.01 | 0.99 (0.89 to 1.09) | 0.8 |
| **Statin use X Lipid ratio** | 1.096 (0.998 to 1.204) | 0.05 | 0.964 (0.851 to 1.092) | 0.6 |
| **Smoking status** |  | <0.001 |  | <0.001 |
| **Never** | 1 |  | 1 |  |
| **Ex** | 0.98 (0.92 to 1.03) |  | 0.87 (0.82 to 0.92) |  |
| **Current** | 1.16 (1.07 to 1.25) |  | 0.97 (0.89 to 1.07) |  |
| **History of alcohol problem (yes v no)** | 1.99 (1.70 to 2.33) | <0.001 | 1.49 (1.15 to 1.25) | 0.003 |
| **History of diabetes (yes v no)** | 1.34 (1.24 to 1.45) | <0.001 | 1.16 (1.07 to 1.25) | <0.001 |
| **History of CHD (yes v no)** | 1.19 (1.12 to 1.28) | <0.001 | 0.97 (0.92 to 1.03) | 0.3 |
| **History of stroke or TIA (yes v no)** | 2.16 (2.01 to 2.32) | <0.001 | 1.37 (1.29 to 1.45) | <0.001 |
| **History of atrial fibrillation (yes v no)** | 1.39 (1.26 to 1.53) | <0.001 | 1.16 (1.08 to 1.25) | <0.001 |
| **Current depression/ use of anti-depressants (yes v no)** | 2.50 (2.35 to 2.66) | <0.001 | 1.67 (1.57 to 1.77) | <0.001 |
| **Current anxiety/use of anxiolytics (yes v no)** | 1.62 (1.46 to 1.80) | <0.001 | 1.34 (1.22 to 1.48) | <0.001 |
| **Current use of hypnotics (yes v no)** | 1.40 (1.27 to 1.54) | <0.001 | 1.11 (1.03 to 1.19) | 0.007 |
| **Current NSAID use excl. aspirin (yes v no)** | 0.94 (0.87 to 1.01) | 0.1 | 0.79 (0.73 to 0.85) | <0.001 |
| **Current aspirin use (yes v no)** | 1.46 (1.38 to 1.55) | <0.001 | 1.15 (1.09 to 1.20) | <0.001 |

*from Cox models, adjusting for age and gender

**Table A.4: Characteristics of sample aged 80-95 years after imputation for variables with missing data at baseline.**

|  | **Development cohort (n = 130,382)** | | **Validation cohort (n = 38,084)** | |
| --- | --- | --- | --- | --- |
|  | **Mean** | **sd** | **Mean** | **sd** |
| **Baseline Total cholesterol, mmol/L** | 5.44 | 1.22 | 5.56 | 1.23 |
| **Baseline HDL cholesterol, mmol/L** | 1.55 | 0.46 | 1.58 | 0.47 |
| **Baseline Weight, kg** | 64.7 | 13.8 | 64.4 | 13.4 |
| **Baseline Systolic blood pressure, mmHg** | 146.6 | 19.9 | 147.2 | 19.8 |
| **Height, m** | 1.61 | 0.10 | 1.62 | 0.10 |
| **Baseline BMI, kg/m2** | 24.9 | 4.55 | 24.6 | 4.43 |
| **Baseline lipid ratio** | 3.73 | 1.14 | 3.76 | 1.14 |
|  |  |  |  |  |
|  | **Freq** | **%** | **Freq** | **%** |
|  |  |  |  |  |
| **Baseline smoking status** |  |  |  |  |
| Never | 74,944 | 57.5 | 23,141 | 60.8 |
| Ex | 43,810 | 33.6 | 11,819 | 31.0 |
| Current | 11,628 | 8.9 | 3,124 | 8.2 |
|  |  |  |  |  |

**Figure A.1: Flowchart of derivation of validation cohort**

**264,224** patients in **95** practices

Exclude:

**3,294** patients with no data on social deprivation

**267,518** patients in **95** practices

Exclude:

**4,981** patients with a history of dementia at baseline

**3,959** patients already experiencing possible prodromal symptoms (memory loss, confusion, cognitive decline)

Exclude:

Patients with a history at baseline of:

Alcohol-induced dementia **n=36**

Drug-induced dementia **n=0**

Dementia in other conditions NOS **n=24**

HIV **n=27**

Huntingdon’s **n=33**

Lewy body disease **n=33**

Parkinson’s **n=4,031**

Pick’s **n=16**

CJD **n=1**

Start: **280,659** patients aged 60 to 95 years in **95** practices

**276,458** patients with no exclusion diagnoses at baseline in **95** practices

**Appendix B. How to calculate the risk of dementia for a new patient from the aged 60-79 years model**

Patient data needed:

| **Predictor** |
| --- |
| Age in **years** |
| Gender |
| Quintile of area deprivation (from patient postal (zip)code)*; in fifths from 1 = least deprived area to 5 = most deprived area) |
| Body mass index in **kg/m^2^** |
| Current use of anti-hypertensive drugs (yes or no) |
| Smoking status in last 5 years (Never/Ex/Current) |
| History of heavy drinking or alcohol problem (yes or no) |
| Current depression and/or use of anti-depressants (yes or no) |
| Current aspirin use (yes or no) |
| History of stroke or TIA (yes or no) |
| History of atrial fibrillation (yes or no) |
| History of diabetes (yes or no) |
| Current calendar year |

*in the UK this is derived by linkages of the postal (zip) code recorded in patient’s notes to UK Population Census data. This is similarly used in other risk calculators in routine use in clinical practice, such as QRisk2 for calculating Cardiovascular Disease risk.

Key to symbols in formula:

| **Symbol** | **Evaluate for patient as follows:** |
| --- | --- |
| age | Age in years |
| I(fem) | Takes value 1 if female; Takes value 0 if male |
| I(t2) | Takes value 1 if patients belongs to Deprivation quintile 2; takes value 0 otherwise |
| I(t3) | Takes value 1 if patients belongs to Deprivation quintile 3; takes value 0 otherwise |
| I(t4) | Takes value 1 if patients belongs to Deprivation quintile 4; takes value 0 otherwise |
| I(t5) | Takes value 1 if patients belongs to Deprivation quintile 5; takes value 0 otherwise |
| bmi | Body mass index in kg/m^2^ |
| I(hyp) | Takes value 1 if use of antihypertensives = yes; takes value 0 if use of antihypertensives = no |
| I(ex) | Takes value 1 if ex smoker; takes value 0 otherwise |
| I(curr) | Takes value 1 if current smoker; takes value 0 otherwise |
| I(alc) | Takes value 1 if patient has history of heavy drinking = yes; takes value 0 if history of heavy drinking = no |
| I(dep) | Takes value 1 if current depression/ use of antidepressants = yes; takes value 0 if no depression/use of antidepressants = no |
| I(asp) | Takes value 1 if use of aspirin = yes; takes value 0 if use of aspirin = no |
| I(str) | Takes value 1 if patient has history of stroke or TIA; takes value 0 if no history of stroke or TIA |
| I(af) | Takes value 1 if patient has history of atrial fibrillation; takes value 0 if no history of atrial fibrillation |
| I(dm) | Takes value 1 if diabetes = yes; takes value 0 if diabetes = no |
| cal | Calendar year e.g. 2013 |

**Formula:**

Define P =

0.20921×(age - 65.608) + -0.00339×(age - 65.608)×(age - 65.608) +

-0.0616×(bmi - 27.501) + 0.002508×(bmi - 27.501)×(bmi - 27.501) +

0.12854×I(fem) + -0.13199×I(hyp) + 0.04477×(cal - 2003.719) +

0.013371×I(t2) + 0.117904×I(t3) + 0.201776×I(t4) + 0.225529×I(t5) +

-0.06792×I(ex) + -0.08657×I(curr) +

0.443535×I(alc) + 0.833612×I(dep) +

0.252833×I(asp) + 0.577207×I(str) + 0.220728×I(af) + 0.286701×I(dm)

Define S = 0.9969

Then predicted 5-year risk (as a percentage) = 100×[1-S^exp(P)^]

**Worked example:**

Take patient X:

| **Predictor** | **Data for patient X** |
| --- | --- |
| Age in **years** | 75 |
| Gender | Female |
| Quintile of deprivation (from patient postcode; in fifths from 1 = least deprived to 5 = most deprived) | 4 |
| Body mass index in **kg/m^2^** | 25 |
| Current use of anti-hypertensive drugs (yes or no) | No |
| Smoking status in last 5 years (Never/Ex/Current) | Never |
| History of heavy drinking or alcohol problem (yes or no) | No |
| Current depression and/or use of anti-depressants (yes or no) | Yes |
| Current aspirin use (yes or no) | No |
| History of stroke or TIA (yes or no) | No |
| History of atrial fibrillation (yes or no) | No |
| History of diabetes (yes or no) | Yes |
| Current calendar year | 2013 |

Calculated % 5-year risk for patient X:

P =

0.20921×(75 - 65.608) + -0.00339×(75 - 65.608)×(75 - 65.608) +

-0.0616×(25 - 27.501) + 0.002508×(25 - 27.501)×(25 - 27.501) +

0.12854×1 + -0.13199×0 + 0.04477×(2013 - 2003.719) +

0.013371×0 + 0.117904×0 + 0.201776×1 + 0.225529×0 +

-0.06792×0 + -0.08657×0 +

0.443535×0 + 0.833612×1 +

0.252833×0 + 0.577207×0 + 0.220728×0 + 0.286701×1

= 0.20921×(75 - 65.608) + -0.00339×(75 - 65.608)×(75 - 65.608) +

-0.0616×(25 - 27.501) + 0.002508×(25 - 27.501)×(25 - 27.501) +

0.12854 + 0.04477×(2013 - 2003.719) +

0.201776 + 0.833612 + 0.286701

= 3.7017581

S = 0.9969

Then predicted 5 year risk that this patient develops dementia in the next 5 years (as a percentage) = 100×[1-S^exp(P)^] = 100×[1-0.9969^exp(3.7017581)^] = **11.8%**
